# Influence of Varied Environment Conditions on the Gut Microbiota of Yaks

**DOI:** 10.3390/ani14111570

**Published:** 2024-05-25

**Authors:** Yanbin Zhu, Jiayi Tian, Yangji Cidan, Hongzhuang Wang, Kun Li, Wangdui Basang

**Affiliations:** 1 Institute of Animal Husbandry and Veterinary Medicine, Tibet Academy of Agriculture and Animal Husbandry Sciences, Lhasa 850009, China; zhuyanbin126@126.com (Y.Z.); 13889092363@163.com (Y.C.); wanghongzhuang66@163.com (H.W.); 2 Linzhou Animal Husbandry and Veterinary Station, Lhasa 850009, China; 3 College of Veterinary Medicine, Gansu Agricultural University, Lanzhou 730070, China; 4 College of Veterinary Medicine, Nanjing Agricultural University, Nanjing 210095, China; 17119104@njau.edu.cn

**Keywords:** yak, gut microbiota, high altitude, temperature, metabolism

## Abstract

**Simple Summary:**

This study investigated the gut microbiota of yaks in the extreme conditions of the Qinghai–Tibetan Plateau. Using amplicon sequencing, 13,683 bacterial and 1912 fungal variants were identified, revealing distinct microbial structures in yaks from different altitudes and temperatures. Firmicutes, Bacteroidota, and Actinobacteriota were dominant bacterial phyla, while Ascomycota and uncultured fungi dominated the fungal community. Certain genera like UCG-005, Christensenellaceae_R-7_group, and Rikenellaceae_RC9_gut_group were prevalent in specific regions. Statistical analysis highlighted significant differences in microbial composition and metabolic functions, particularly in digestive systems at different altitudes. Notably, microbial diversity, richness, and specific genera varied across regions, suggesting the adaptability of yak gut microbiota to high-altitude conditions. This study sheds light on the significant impact of altitude and temperature on yak gut microbiota, offering valuable insights into their adaptability in extreme environments.

**Abstract:**

Despite the crucial role of the gut microbiota in different physiological processes occurring in the animal body, reports regarding the gut microbiota of animals residing in different environmental conditions like high altitude and different climate settings are limited. The Qinghai–Tibetan Plateau is renowned for its extreme climatic conditions that provide an ideal environment for exploring the effects of high altitude and temperature on the microbiota of animals. Yaks have unique oxygen delivery systems and genes related to hypoxic response. Damxung, Nyêmo, and Linzhou counties in Tibet have variable altitudes and temperatures that offer distinct settings for studying yak adaptation to elevated terrains. The results of our study suggest that amplicon sequencing of V3-V4 and internal transcribed spacer 2 (ITS2) regions yielded 13,683 bacterial and 1912 fungal amplicon sequence variants (ASVs). Alpha and beta diversity indicated distinct microbial structures. Dominant bacterial phyla were Firmicutes, Bacteroidota, and Actinobacteriota. Genera UCG-005, Christensenellaceae_R-7_group, and Rikenellaceae_RC9_gut_group were dominant in confined yaks living in Damxung county (DXS) and yaks living in Linzhou county (LZS), whereas UCG-005 prevailed in confined yaks living in Nyêmo county (NMS). The linear discriminant analysis effect size (LEfSe) analysis highlighted genus-level differences. Meta-stat analysis revealed significant shifts in bacterial and fungal community composition in yaks at different high altitudes and temperatures. Bacterial taxonomic analysis revealed that two phyla and 32 genera differed significantly (*p* < 0.05). Fungal taxonomic analysis revealed that three phyla and four genera differed significantly (*p* < 0.05). Functional predictions indicated altered metabolic functions, especially in the digestive system of yaks living in NMS. This study reveals significant shifts in yak gut microbiota in response to varying environmental factors, such as altitude and temperature, shedding light on previously unexplored aspects of yak physiology in extreme environments.

## 1. Introduction

Animal microbiomes are considered extensions of the host’s physiology, anatomy, and even genomic structure [1]. When examined across different animals, the composition of the animal biome varies significantly [2]. The gut microbiota, also known as the “second genome”, is a complex and dynamic system that co-evolves with the host [3,4]. Microorganisms including bacteria, archaea, fungi, and viruses encode over three million genes generating thousands of metabolites [5,6]. The gut microbiota actively engages in numerous physiological processes that include the digestion of food, regulation of the immune system, and preservation of the intestinal mucosal barrier [7]. Microbes that are produced through the fermentation of undigested food craft essential short-chain fatty acids and contribute not only to providing energy but also to the well-being of the intestinal mucous membrane [8]. These microbial inhabitants exert their influence on the host’s immune system acting as gatekeepers to suppress the growth of harmful microorganisms and maintain a balanced microbial equilibrium in the gut [9]. Recent reports have highlighted the connection between the composition of the rumen microbiome and economically significant traits that include feed efficiency and methane emissions [10]. Multiple factors such as pregnancy, early milk feeding, water intake, initial dietary provisions, prebiotics, and genetic modifications exert considerable impact on the colonization and maturation of the rumen microbiota [11]. However, limited studies have delved into the structure and functionality of the gut microbiota in high-altitude animals [12].

The Qinghai–Tibetan Plateau is also known as the “roof of the world”; this region experiences intense radiation, abundant sunshine, low temperatures, limited heat accumulation, and a significant decrease in temperature with increasing altitude [13,14]. The three counties in Tibet have different environmental conditions influenced by altitude and temperature. Damxung county is located at east longitude 90°45′–91°31′ and northern latitude 29°31′–31°04′, with an average elevation of 4200 m, temperature of 1.3 °C, frost-free period of 62 days, rainfall of 456.8 mm, evaporation 1725.7 mm, and sunshine of 2880.9 h [15]. Linzhou county is located at east longitude 90°51′–91°28′ and northern latitude 29°45′–30°08′, with an average elevation of 4200 m and an annual average temperature of 7.5 °C; during January the temperature drops to approximately −5.4 °C, with extreme lows below −22 °C. The frost-free period in Linzhou county extends for 120 days, annual rainfall is 491 mm, and sunshine is 3000 h [15,16]. Nyêmo county is located at east longitude 90.1° and northern latitude 29.4°, at an altitude above 3800 m; this region experiences an annual average temperature of 6.7 °C, with temperatures reaching −40 °C in January and 15.0 °C in July. The annual precipitation of Nyêmo county is 295.3 mm, the frost-free period extends for 100 days, and the average amount of sunshine is 2947.8 h. Yaks in Tibet achieve adaptation to high-altitude environments by establishing a sophisticated oxygen delivery system participating in three physiological processes: oxygen intake, transportation, and utilization [17,18]. Moreover, at the genomic level, yaks harbor positively selected genes involved in hypoxic response and energy metabolism [19,20]. As a ruminant with a distinctive rumen microbiota, yaks provide an excellent opportunity for studying the impact of altitude and temperature on the intestinal microbial community of these animals [21].

Recent advancements in genome sequencing technologies, bioinformatics, and culturomics enable researchers to explore microbial communities in greater detail than ever [22]. The in-depth exploration of the gut microbiota provides researchers with an opportunity to better understand the intricate interactions between the microbial ecosystem and the host [23]. Previous studies reported microbiota differences in different plateau ruminant breeds [24] and yaks in different seasons [25]; however, little information is available about the microflora of yaks from different altitude and temperature regions. Therefore, this study aims to explore the effects of altitude and temperature on the intestinal microflora of yaks by sequencing yak feces from three different counties in Tibet.

## 2. Materials and Methods

### 2.1. Sample Collection

A total of 18 six-year Pagri yaks having the same weight (400–410 kg) and age were chosen as samples from Damxung, Linzhou, and Nyêmo counties in Tibet, China (6 samples per region). The yaks were mainly free-ranged and received additional supplements with some oats and alfalfa (1:1) daily. In April 2023, the temperature range in Damxung was −5–10 °C, Linzhou was −1–15 °C, and Nyêmo was −9–16 °C. The samples were divided into three groups: yaks raised through confined feeding in Damxung county (DXS), and those in Linzhou county (LZS) and Nyêmo county (NMS). Approximately 200 g of fresh rectal feces were collected from each chosen yak using a sampler. The fresh fecal samples obtained were sub-sampled from the central portion and the final samples were rapidly frozen using liquid nitrogen and stored at −80 °C for further analysis.

### 2.2. DNA Extraction and 16S rDNA Amplicon Sequencing

According to the manufacturer’s instructions, total genome DNA from 18 samples were extracted using the OMEGA Soil DNA Kit (D5625-01) (Omega Bio-Tek, Norcross, GA, USA). A 1% agarose gel was used to test the purity and concentration of the obtained DNA so that total genome DNA could be guaranteed.

According to the selected sequencing regions, the universal primers 338F: 5′-ACTCCTACGGGAGGCAGCA-3′ and 806R: 5′-GGACTACHVGGGTWTCTAAT-3′ were used for amplifying V3-V4 variable regions of bacterial 16S rDNA genes. Also, the universal primers ITS5F: 5′-GGAAGTAAAAGTCGTAACAAGG-3′ and ITS1R: 5′-GCTGCGTTCTTCATCGATGC-3′ were used for amplifying ITS2 regions [26,27]. The amplified Polymerase Chain Reaction (PCR) products were detected by 2% agarose gel electrophoresis and the target fragments were cut and recovered by the Quant-iT PicoGreen dsDNA Assay Kit (Thermo Fisher Scientific, Waltham, MA, USA)for purification of the product [28].

Referring to the preliminary quantitative results of electrophoresis, the products recovered by Polymerase Chain Reaction (PCR) amplification were detected and quantified with a Microplate reader (BioTek, Winooski, VT, USA) fluorescence quantitative system, and corresponding proportions were mixed according to the sequencing requirements of each sample.

Following the manufacturer’s recommendations and index codes, a library was constructed using the TruSeq Nano DNA LT Library Prep Kit (Illumina, San Diego, CA, USA) [29]. After being inspected by the Agilent Bioanalyzer 2100 (Agilent Technologies Co Ltd., Santa Clara, CA, USA) and Promega QuantiFluor (Promega Corporation, Madison, WI, USA), the library was further qualified and sequenced.

### 2.3. Bioinformatics and Statistical Analysis

The raw data in Fastq Sequence and Quality Format (FASTQ) obtained from sequencing contain a certain proportion of interference (Dirty Data). In order to improve the accuracy and reliability of the information analysis, paired-end reads were preprocessed using Cutadapt software (1.9.1) to detect and cut off the adapt [30]. Then, the raw data were subjected to quality filtering (reads < 200 bp and low quality), denoising, merging, and dechimerization using the Divisive Amplicon Denoising Algorithm (DADA2) with the default parameters of Quantitative Insights Into Microbial Ecology (QIIME2) [31,32]. Sequences with an abundance of less than 10 were filtered out resulting in the generation of amplicon sequence variants (ASVs). Lastly, the software output the clean data, and the ASV abundance table was obtained.

Venn diagrams were generated to show common and unique ASVs among the three groups. For the purpose of evaluating sequencing depth and data volume, the rarefaction curves and Shannon curves were drawn.

The alpha diversity index, which contained an Abundance-based Coverage Estimator (ACE), Chao1 richness estimator (Chao1), Good’s coverage, Observed Species, PD whole_tree, Shannon and Simpson indexes, was calculated by QIIME2 software (v2023.5) to analyze the diversity of the microbial communities within the samples [33]. The beta diversity was also detected to compare microbial community composition between different samples, including principal coordinate analysis (PCoA), which assessed the similarities and differences among different groups.

Moreover, differential bacteria at both phylum and genus levels were identified using Metastats analysis, linear discriminant analysis effect size (LEfSe), and Fisher’s least significant difference (LSD) score.

The potential metabolic function of flora was predicted with Phylogenetic Investigation of Communities by Reconstruction of Unobserved States (PICRUSt2), which could accurately predict the abundance of host-associated and environmentally relevant gene family communities with quantifiable uncertainty [34].

### 2.4. Statistical Analysis

Obtained amplicon sequencing data were analyzed by using GraphPad Prism (v8.0) and the SPSS statistical program (v20.0). Values with *p* < 0.05 (means ± SD) were considered statistically significant.

## 3. Results

### 3.1. Microbial Diversity

Different altitudes and temperatures affect the gut bacterial and fungi microbial structure diversities of yaks living in different environments.

The raw sequences of V3-V4 and internal transcribed spacer 2 (ITS2) regions of 18 fecal samples were acquired after amplicon sequencing. A total of 2,546,119 (DXS = 857,146, LZS = 845,720, and NMS = 843,253) and 2,520,546 (DXS = 839,547, LZS = 841,517, and NMS = 839,482) sequences from V3-V4 and ITS2 regions of all three groups were obtained (Table 1). To ensure the results of data analysis, raw data were filtered and a total of 2,440,372 (DXS = 820,954, LZS = 812,162, and NMS = 807,256) and 2,422,604 (DXS = 799,849, LZS = 807,887, and NMS = 814,868) filtered sequences were obtained.

**Figure 1:**
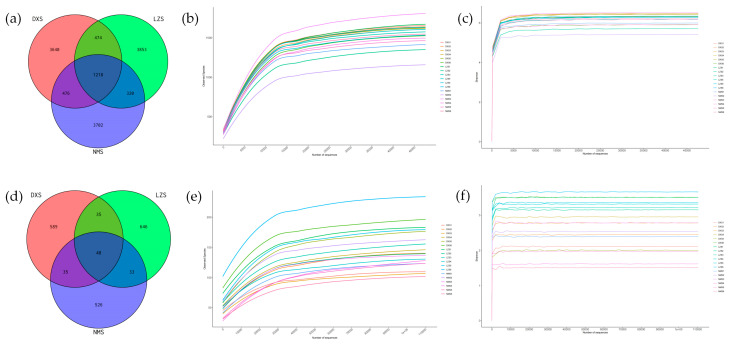
(**a**) Gut bacterial ASV distribution in different samples; (**b**) bacterial rarefaction curves for all samples; (**c**) bacterial Shannon curves for all samples; (**d**) gut fungal ASV distribution in different samples; and (**e**,**f**) fungal rarefaction curves and Shannon curves for all samples.

Amplicon sequence variants (ASVs) were recognized by using DADA2 in QIIME2 for denoising raw reads and conducting dereplication. The sequences were clustered into 13,683 ASVs in total out of which 3640, 3853, and 3702 ASVs were unique in the DXS, LZS, and NMS groups individually. In the fungal community, the sequences were clustered into 1912 ASVs of which there were 83 ASVs shared between the DXS and LZS groups. A total of 83 ASVs shared between the DXS and NMS groups and 81 ASVs were shared between the LZS and NMS groups (Figure 1a,d). Through rarefaction and Shannon curves, the tendency of flattening was shown, which indicated that the sequencing data volume is sufficient and the sequencing depth meets the requirements (Figure 1b,c,e,f). Therefore, the sequencing results were adequate to reflect the biodiversity contained in the current samples.

The multiple alpha and beta diversity indices were calculated to describe the alteration in the intestinal microflora in yaks living in different altitudes and temperatures.

**Figure 2:**
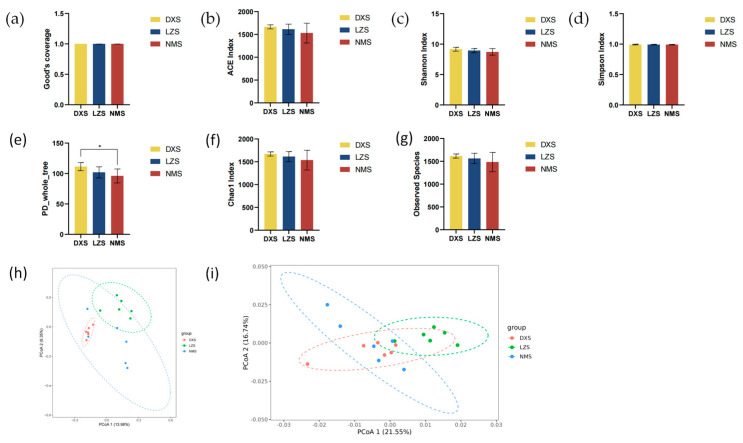
Gut bacterial diversities. (**a**) Good’s coverage index; (**b**) ACE index; (**c**) Shannon index; (**d**) Simpson index; (**e**) PD_whole_tree; (**f**) Chao1 index; (**g**) Observed Species index; (**h**) PCoA analysis based on unweighted distance; and (**i**) PCoA analysis based on weighted distance. All of the data represent means ± SD. * *p* < 0.05.

**Figure 3:**
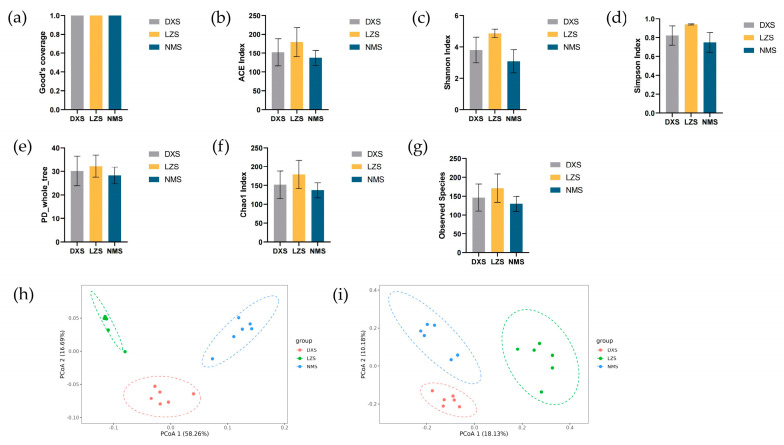
Gut fungal diversities. (**a**) Good’s coverage index; (**b**) ACE index; (**c**) Shannon index; (**d**) Simpson index; (**e**) PD_whole_tree index; (**f**) Chao1 index; (**g**) Observed Species index; (**h**) PCoA analysis based on unweighted distance; and (**i**) PCoA analysis based on weighted distance.

In alpha diversity analysis, the Good’s coverage of the three groups in both bacterial and fungal populations ranged from 99.7% to 99.82% and 99.98% to 99.99%, illustrating that the sampling is relatively sufficient with a considerable number of discovered species and it is likely approaching the actual species diversity of the entire ecosystem (Figure 2a and Figure 3a).

Moreover, statistical analysis demonstrated that the bacterial ACE index (1665.86 ± 44.37 versus 1612.7 ± 112.4 versus 1528.86 ± 216.71, *p* = 0.3065), Shannon index (9.16 ± 0.32 versus 8.93 ± 0.35 versus 8.72 ± 0.54, *p* = 0.2588), Simpson index (0.99 ± 0.01 versus 0.99 ± 0.01 versus 0.99 ± 0.01, *p* = 0.7148), Chao1 index (1671.71 ± 46.58 versus 1613.16 ± 110.12 versus 1536.18 ± 216.82, *p* = 0.2580) and Observed Species index (1615.17 ± 45.03 versus 1563.33 ± 113.57 versus 1483 ± 210.16, *p* = 0.3232), as well as the fungal ACE index (152.20 ± 32.77 versus 179.22 ± 35.40 versus 137.49 ± 18.34, *p* = 0.12), Observed Species index (146.17 ± 32.41 versus 171.00 ± 34.48 versus 129.67 ± 18.25, *p* = 0.096), and PD_whole_tree index (30.14 ± 5.76 versus 32.23 ± 4.28 versus 28.27 ± 3.23, *p* = 0.37), were not significantly different between the three groups (Figure 2b–d,f,g and Figure 3b,e,g). However, the bacterial PD_whole_tree index (111.31 ± 6.03 versus 101.91 ± 8.35 versus 96.01 ± 10.44, *p* = 0.034), fungal Shannon index (3.80 ± 0.74 versus 4.86 ± 0.25 versus 3.08 ± 0.68, *p* = 0.0068), Simpson index (0.82 ± 0.19 versus 0.94 ± 0.01 versus 0.75 ± 0.10, *p* = 0.0023), and Chao1 index (151.82 ± 33.34 versus 179.16 ± 34.33 versus 137.37 ± 18.23, *p* = 0.076) exhibited significant differences among the three groups, implying that the intestinal microbiota changed (Figure 2e and Figure 3c,d,f).

In beta diversity analysis, the principal coordinate analysis (PCoA) could evaluate the differences and similarities between and within groups. On the basis of weighted and unweighted infraction of intestinal flora, PCoA analysis revealed that dots in the DXS, LZS, and NMS groups were separated, demonstrating the fact that different altitudes and temperatures affect the gut microbial structure of yaks living in different environments (Figure 2h,i and Figure 3h,i).

### 3.2. Intestinal Bacterial Composition Analysis

Different altitudes and temperatures affect the gut bacterial microbial structure of yaks living in different environments.

**Figure 4:**
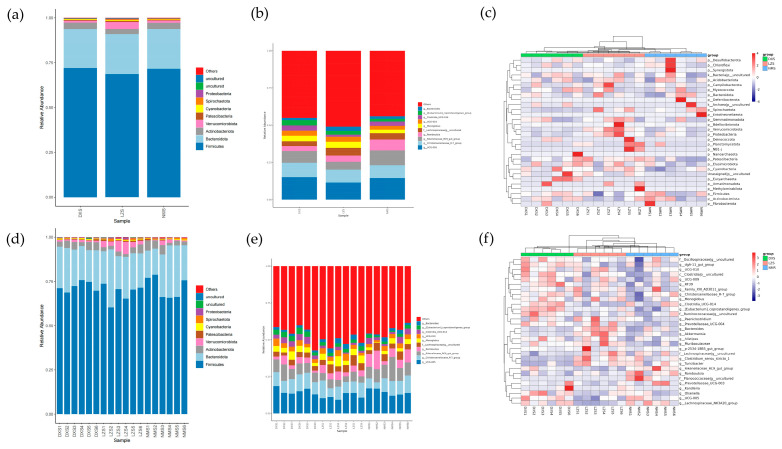
The relative abundances and distribution of regnant bacteria in the DXS, LZS, and NMS groups. (**a**,**d**) The gut bacterial composition of each group and every sample at the phylum level; (**b**,**e**) gut bacterial composition of each group and every sample at the genus level; (**c**,**f**) clustering heatmap of yak living in different altitude and temperature conditions at the phylum and genus levels. The color values of the heatmap indicate the normalized relative richness of each species.

Microbial classification methods were used to determine the relative abundance of dominant taxa at the levels of phylum and genus. At the phylum level, 36 phyla were detected in the gut microbial community ranging from 15–20 per sample. As shown in the bar chart of the cumulative relative abundance of the top 10 species at phylum and genus levels, the phyla Firmicutes (DXS = 72.09%, LZS = 68.62%, and NMS = 71.61%), Bacteroidota (DXS = 21.53%, LZS = 22.25%, and NMS = 22.11%), and Actinobacteriota (DXS = 3.73%, LZS = 2.82%, and NMS = 3.63%) were the three most dominant phyla in the DXS and NMS groups, comprising about 97.35% of the bacterial community. The third largest proportion of the LZS group was Verrucomicrobiota (3.92%), which comprised only 1.07% in the DXS group and 1.08% in the NMS group. Other phylum such as Patescibacteria (DXS = 0.46%, LZS = 0.69%, and NMS = 0.32%), Cyanobacteria (DXS = 0.43%, LZS = 0.44%, and NMS = 0.28%), Spirochaetota (DXS = 0.09%, LZS = 0.48%, and NMS = 0.43%), and Proteobacteria (DXS = 0.22%, LZS = 0.46%, and NMS = 0.26%) were identified with lower abundance (Figure 4a,d).

To further assess the changes in gut bacterial compositions in yaks living in different altitudes and temperature environments, a total of 493 genera were discovered. At the genus level, UCG-005 (DXS = 15.12%, LXS = 11.49%), Christensenellaceae_R-7_group (DXS = 9.62%, LZS = 8.71%), and Rikenellaceae_RC9_gut_group (DXS = 7.84%, LZS = 5.08%) were the three most dominant genera in the DXS group as well as the LZS group. Moreover, UCG-005 (14.54%) was the dominant genus in the NMS group, followed by Rikenellaceae_RC9_gut_group (9.95%) and Christensenellaceae_R-7_group (8.58%), comprising about 33.07% of the gut microbial composition. Other genera’s proportions such as Romboutsia (DXS = 3.22%, LZS = 4.30%, and NMS = 7.29%), Monoglobus (DXS = 3.68%, LZS = 4.22%, and NMS = 2.35%), and UCG-010 (DXS = 3.29%, LZS = 3.27%, and NMS = 2.64%) were relatively small (Figure 4b,e).

Furthermore, the clustering heatmap representing the distribution and diversity of the gut bacterial community at the phylum and genus levels was employed to aid in visually identifying species that are more or less abundant within specific groups among the three groups (Figure 4c,f).

**Figure 5:**
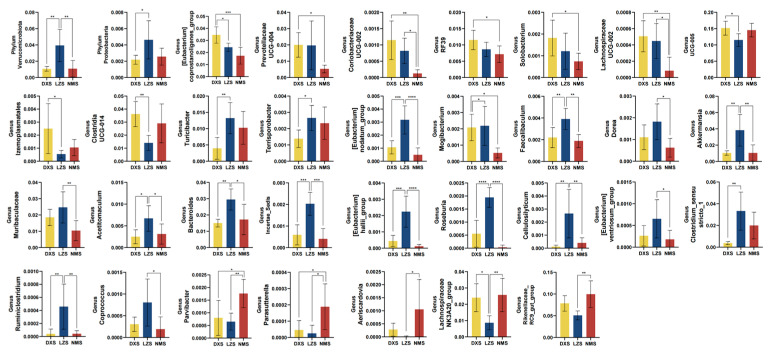
The gut bacterial comparisons among the DXS, LZS, and NMS groups at the phylum and genus levels. Metastats analysis was applied to identify the significantly differentially abundant bacterial genera among the three groups and all of the data represent means ± SD. * *p* < 0.05, ** *p* < 0.01, *** *p* < 0.001, **** *p* < 0.0001.

Metastats analysis was used to further compare the differences in the gut bacterial community among the DXS, LZS, and NMS groups. At the phylum level, Verrucomicrobiota was significantly decreased in the DXS and NMS groups. Also, Proteobacteria was reduced in the DXS and NMS groups but only in the DXS group was it significantly different (*p* < 0.05). Moreover, at the genus level, 32 bacterial genera were analyzed to be statistically significant. Among them [Eubacterium]coprostanoligenes group, Prevotellaceae_UCG-04, Coriobacteriaceae_UCG-002, Solobacterium, Lachnospiraceae_UCG-002, UCG-005, Clostridia_UCG-014, and Izemoplasmatales accounted for the highest proportion in the DXS group compared to the other two groups. Meanwhile, these bacteria including Turicibacter, Terrisporobacter, Muribaculaceae, Mogibacterium, Faecalibaculum, Dorea, Akkermansia, Acetitomaculum, [Eubacterium]_nodatum_group, Bacteroides, Incertae_Sedis, Roseburia, [Eubacterium]_hallii_group, Coprococcus, Cellulosilyticum, Clostridium_sensu_stricto_1, Ruminiclostridium, and [Eubacterium]_ventriosum_group had the highest relative abundance in the LZS group compared to the DXS and NMS groups. The five genera exhibited the highest prevalence within the NMS group and one of them was Aeriscardovia, which was barely detected in the LZS group (Figure 5).

**Figure 6:**
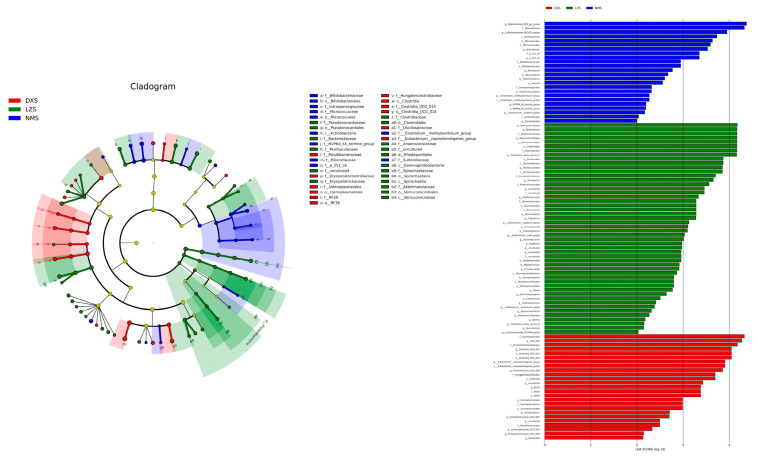
Integrated linear discriminant analysis effect size (LEfSe) analysis and LDA scores of the gut bacterial microbiota among the DXS, LZS, and NMS groups demonstrated the different taxa related to various altitude and temperature conditions. The criterion of significance was performed at LDA scores > 2.

In addition, the linear discriminant analysis effect size (LEfSe) results (LDA score > 2), which are commonly referred to as biomarkers, further identified microorganisms with elevated abundance in each group compared to the other groups (Figure 6).

### 3.3. Intestinal Fungal Composition Analysis

Different altitudes and temperatures affect the gut fungi microbial structure of yaks living in different environments.

**Figure 7:**
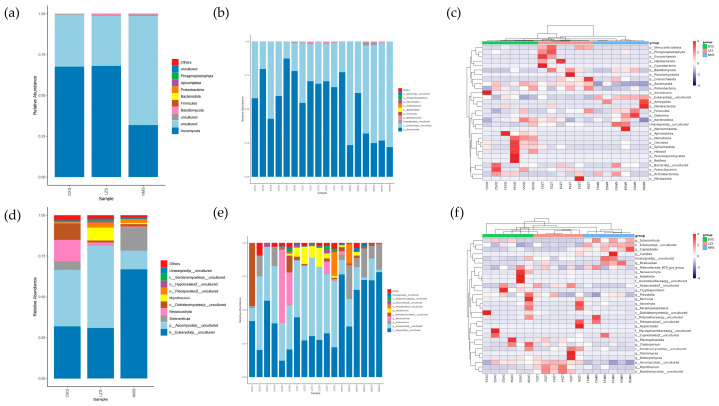
The relative abundances and distribution of regnant fungus in the DXS, LZS, and NMS groups. (**a**,**d**) Gut fungal composition of each group and every sample at the phylum level; (**b**,**e**) gut fungal composition of each group and every sample at the genus level; (**c**,**f**) clustering heatmap of yak living in different altitude and temperature conditions at the phylum and genus levels. The color values of the heatmap indicate the normalized relative richness of each species.

At the phylum level, 39 phyla were observed in the intestinal fungal community ranging from 8 to 16 per sample. The phylum Ascomycota (DXS = 67.61%, LZS = 68.01%, and NMS = 31.82%) was the dominant phylum in both the DXS and LZS groups, whereas uncultured phyla (DXS = 31.89%, LZS = 30.87%, and NMS = 66.89%) were present abundantly in the NMS group. Both Ascomycota and some uncultured phyla accounted for over 98% of total fungal taxa (Figure 7a,d). At the genus level, 196 genera were identified. In the bar chart of the cumulative relative abundance of the top 10 species at the genus level, Neoascochyta (DXS = 13.12%, LZS = 1.68%, and NMS = 0.45%) had the highest abundance in the DXS group among all three groups, Myrothecium (DXS = 0.27%, LZS = 7.84%, and NMS = 0.70%) dominated in the LZS group, and Scleromitrula (DXS = 5.21%, LZS = 0.18%, and NMS = 14.27%) took a leading edge in the NMS group (Figure 7b,e). The distribution and comparative abundance of identified fungal genera were further explored through clustering analysis as depicted in the heatmap (Figure 7c,f).

**Figure 8:**
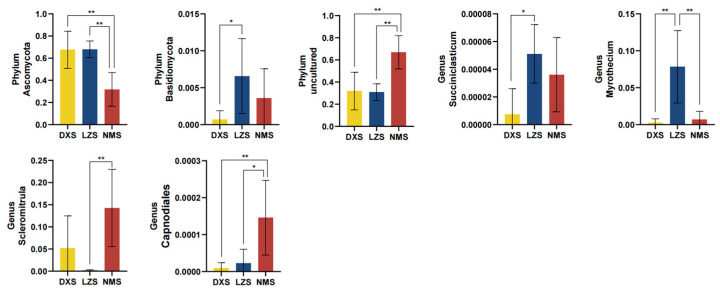
The gut fungal comparisons among the DXS, LZS, and NMS groups at the phylum and genus levels. Metastats analysis was applied to identify the significantly differentially abundant bacterial genera among the three groups and all of the data represent means ± SD. * *p* < 0.05, ** *p* < 0.01.

**Figure 9:**
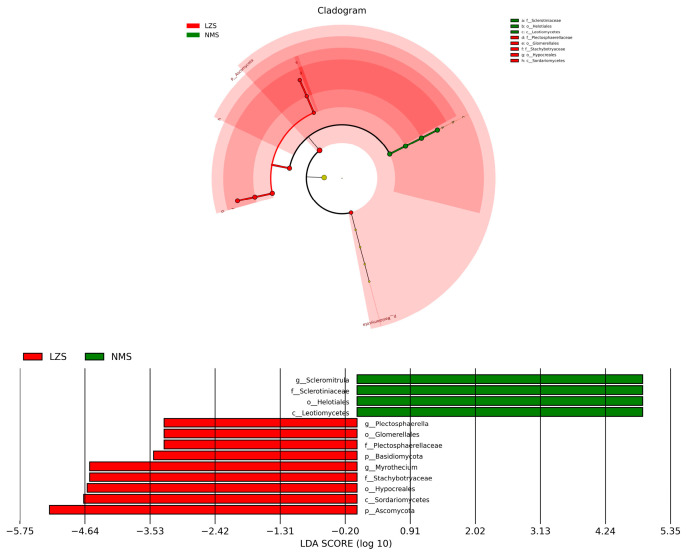
Integrated linear discriminant analysis effect size (LEfSe) analysis and LDA scores of the gut fungal microbiota among the DXS, LZS, and NMS groups demonstrated the different taxa related to various altitude and temperature conditions. The criterion of significance was performed at LDA scores > 2.

By applying Metastats analysis, shifts in the fungal community were fully revealed. At the phylum level, compared to the DXS and LZS groups, Ascomycota decreased a lot in the NMS group. The LZS group had the most relative abundance of Basidlomycota and differed significantly from the DXS group. Moreover, uncultured phyla predominated in the NMS group. Furthermore, at the genus level, four genera were found to be statistically different. Succiniclasticum and Myrothecium occupied the largest proportion in the LZS group, whereas the genera Scieromitrula and Capnodiales had the highest relative abundance in the NMS group (Figure 8). The LEfSe analysis and LDA scores (>2) were applied to better describe the alterations among the three groups (Figure 9).

### 3.4. Metabolic Function Prediction

Different altitude and temperature conditions affect the gut microbial function of yaks living in different environments.

Using PICRUSt2 software (v2.3.0 beta), the species composition information obtained by aligning 16S sequencing data was utilized to infer the functional gene composition in the samples in order to analyze differences among the three groups.

**Figure 10:**
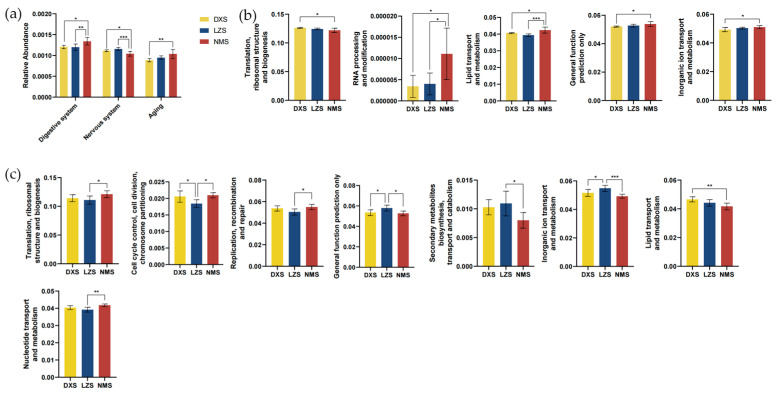
(**a**) Bacterial KEGG function prediction analysis; (**b**) bacterial COG function prediction; and (**c**) fungal COG function prediction. All of the data represent means ± SD. * *p* < 0.05, ** *p* < 0.01, *** *p* < 0.001.

The bacterial metabolism analysis revealed that in the Kyoto Encyclopedia of Genes and Genomes (KEGG) functional prediction analysis, the relative abundance of the digestive system significantly increased in the NMS group (Figure 10a). In the Clusters of Orthologous Groups of proteins (COGs) functional prediction, the relative abundance of “Translation, ribosomal structure and biogenesis”, “General function prediction only”, “Inorganic ion transport and metabolism”, “Lipid transport and metabolism”, and “RNA processing and modification” were significantly different (Figure 10b). Similarly, within the fungal COGs functional prediction, significant differences were observed in the relative abundance of key categories such as “Inorganic ion transport and metabolism”, “Nucleotide transport and metabolism”, “Lipid transport and metabolism”, “General function prediction only”, “Cell cycle control, cell division, chromosome partitioning”, “Secondary metabolites biosynthesis, transport, and catabolism”, “Replication, recombination and repair”, and “Translation, ribosomal structure, and biogenesis” among the three groups (Figure 10c). These findings suggest that the adaptation to environmental changes led to alterations in metabolic functions.

## 4. Discussion

The diversity and abundance of the gut microbiota are crucial in maintaining an ecological balance within the intestinal tract [35,36]. However, there is a scarcity of reports related to variations in the gut microbiota of yaks residing in diverse altitudes and temperatures. Combined with the substantial quantity and intricate composition of gut microbiota, research in this area presents challenges. With the advancement of high-throughput sequencing technologies, 16S rDNA sequencing has emerged as a pivotal tool for investigating the intricacies of gut microbiota [37]. In this study, we applied high-throughput 16S rDNA sequencing to scrutinize the impact of varying altitudes and temperatures on the gut microbiota of yaks that were native to three distinct counties in Tibet. Among the selected three counties, Nyêmo county has an altitude of 3800 m, which is lower compared to the altitudes of 4200 m in Damxung county and Linzhou county [38]. Moreover, Linzhou county has the highest average annual temperature at 7.5 °C, while Damxung county experiences the lowest average annual temperature at 1.3 °C, and Nyêmo county has an annual temperature of 6.7 °C [39,40].

There are discernible variations in the diversity and composition of gut microbiota among humans and other mammals in regions with different altitudes [41]. It has been reported that after acclimatization to high-altitude environments, mice experience a 115-fold increase in the total number of anaerobic bacteria in the gastrointestinal tract whereas the aerobic bacteria decreased 50 times [42]. Furthermore, the gut microbiota of ruminants, rodents, and humans undergo changes corresponding to alterations in altitude [43]. These findings collectively suggest the influence of diverse altitudes on the gut microbiota of yaks. The gut microbiota of white-lipped deer during the withering grassy season was significantly affected when the temperature was higher [44]. Another study revealed that the alpha diversity of musk deer’s intestinal flora—specifically focusing on Firmicutes to Bacteroidetes (FMD) and Actinobacteria to Bacteroidetes (AMD)—was higher during the cold season compared to the warm season, suggesting that seasonal variations have a significant impact on the diversity of musk deer’s intestinal flora [45]. These studies suggest that changes in temperature affect gut microbiota.

Chao1, ACE, and Observed Species indices were applied for the assessment of species richness, while Shannon, PD_whole_tree, and Simpson indices provided insights into species diversity among the alpha diversity analysis [46]. Elevated Chao1, ACE, and Observed Species indices signify increased richness, whereas elevated Shannon and PD_whole_tree indices and a reduced Simpson index indicate high diversity [47]. In this study, the microbial diversity and richness of intestinal bacteria of yaks living in Damxung county were higher than those living in Linzhou county, which was higher than those living in Nyêmo county. Moreover, the yak population in Linzhou county exhibited greater diversity and richness of fungal gut microbes compared to the two other groups.

The community structure components of intestinal microflora of yaks living in Damxung, Nyêmo, and Linzhou counties were different. The research indicated that in yaks raised in the three counties, the most dominant bacterial phyla were Firmicutes and Bacteroidetes regardless of the altitude and temperature of the environment. This was consistent with previous research that analyzed the microbial community characteristics across different segments of the yak intestinal tract. The findings of the earlier study concluded that the predominant bacterial phyla were Firmicutes, Bacteroidetes, and Proteobacteria [48]. Furthermore, these leading phyla were also found in other ruminants such as Mongolian cattle and goats [49,50]. Metagenomics research has revealed that bacteria belonging to the phylum Firmicutes were enriched with genes related to biosynthesis and membrane transport [51]. Moreover, they had the capacity to produce various B-group vitamins and might be associated with anti-inflammatory effects and improvements in intestinal barrier function [52]. On the other hand, bacteria of the phylum Bacteroidetes could utilize a variety of dietary soluble polysaccharides and possess genes for the production of vitamins and coenzymes [53,54]. Both phyla play crucial roles in the digestion and nutrient utilization within the intestinal microbiota of ruminant animals. The research by Jiang et al. indicated that during the cold season, there was an elevated presence of Firmicutes, and the F/B ratio was higher compared to the warm season [45]. This is beneficial to yaks accustomed to high altitude, low oxygen, and low-temperature environments.

To further understand the alteration in the gut microbiota of yaks living in different altitude and temperature conditions in Tibet, significantly different microbial species were discovered at both phylum and genus levels by using Metastats analysis. Compared to others at the phylum level, Verrucomicrobiota and Proteobacteria were abundant in samples obtained from yaks residing in Linzhou county where the altitude is 4200 m and the average temperature is 7.5 °C. The phylum Verrucomicrobia was a group with few cultured representative species and was found in diverse environments such as oceans, lakes, soils, and the gastrointestinal tracts of both animals and humans [55]. As the only cultivated intestinal representative of the Verrucomicrobia, Akkermansia had been observed in patients with inflammatory bowel diseases, particularly ulcerative colitis and metabolic disorders, suggesting that it has potential anti-inflammatory properties [56]. Also, within the mucous layer of the gastrointestinal tract, Akkermansia could establish colonization, activate mucosal microbial networks, enhance intestinal barrier function, and contribute to essential host immune responses [57]. As a bacterial phylum containing some opportunistic pathogens such as Salmonella and Escherichia coli, the abundance of Proteobacteria is susceptible to environmental factors, and its fluctuations often impact the health status of the host [58]. In colons of newborn healthy mammals, Proteobacteria tend to be more abundant, primarily serving to absorb oxygen and create an anaerobic environment that is conducive to the colonization of obligate anaerobes. In colons of healthy adult mammals, the abundance of Proteobacteria is lower, which assists the host in maintaining an anaerobic environment in the intestines [35]. Intestinal inflammation, antibiotics, diet, and genetic factors are responsible for the increased abundance of Proteobacteria [59]. It has been demonstrated that in the gastrointestinal tract of yak, the phylum Proteobacteria is in abundance and plays a significant role in fulfilling the substantial nutrient and energy needs of yak [60].

In terms of gut bacteria, at the genus level, samples collected from yaks living in Damxung county, where the altitude is 4200 m and the average temperature is 1.3 °C, exhibited richness in [Eubacterium]_coprostanoligenes_group, Prevotellaceae_UCG-04, Coriobacteriaceae_UCG-002, Solobacterium, Lachnospiraceae_UCG-002, UCG-005, Clostridia_UCG-014, and Izemoplasmatales. In the oral cavity, Solobacterium contributes to the balance of the oral ecosystem and interacts with other oral bacteria [61]. In the intestinal tract, the functions of Solobacterium are gradually being unveiled through ongoing research. The intestinal microbiota plays a crucial role in host health and immune systems. Solobacterium is suggested to be involved in intricate networks of interactions. Solobacterium in the intestinal tract is associated with host metabolism and immune regulation but the specific mechanisms of its actions require further research for confirmation [62]. Moreover, research suggests that Izemoplasmatales play a role in providing protection against invading viruses, inhibiting the colonization and growth of harmful bacteria, and other related functions [63]. Meanwhile, Turicibacter, Terrisporobacter, Muribaculaceae, Mogibacterium, Faecalibaculum, Dorea, Akkermansia, Acetitomaculum, [Eubacterium]_nodatum_group, Bacteroides, Incertae_Sedis, Roseburia, [Eubacterium]_hallii_group, Coprococcus, Cellulosilyticum, Clostridium_sensu_stricto_1, Ruminiclostridium, and [Eubacterium]_ventriosum_group were enriched in the samples collected from yak residing in Linzhou county. Bacteria belonging to the Turicibacter genus are vital members of the mammalian intestinal microbiota. They are associated with changes in dietary fat and body weight along with having the ability to modulate host bile acid and lipid metabolism genes [64]. Studies suggest a direct association between elevated abundance of Terrisporobacter and an increased risk of sepsis [65]. Moreover, Muribaculaceae is linked to the regulation of the gut microbiota, mitigating sepsis-related liver damage [66]. Furthermore, samples collected from yaks residing in Nyêmo county, where the altitude is 3800 m and the average temperature is 6.7 °C, exhibited a relatively high abundance of five genera including Parasutterella, Aeriscardovia, Lachnospiraceae_NK3A20_group, Rikenellaceae_RC9_gut_group, and Parvibacter. Parasutterella is believed to play a potential role in preserving bile acid homeostasis and cholesterol metabolism, with isolates from mice demonstrating anti-glycolytic properties [67]. Aeriscardovia plays a significant role in preventing intestinal infections, reducing cholesterol levels, improving serum antioxidant capacity, immune modulation, and maintaining intestinal health [68].

In terms of gut fungus at the phylum level, samples collected from yaks living in Damxung and Linzhou counties had a higher abundance of Ascomycota while Basidiomycota had the largest proportion of yaks living in Linzhou county. At the genus level, Succiniclasticum and Myrothecium dominated the LZS group, constituting the majority, whereas the genera Scieromitrula and Capnodiales exhibited the highest relative abundance in the NMS group. Succiniclasticum has the ability to quantitatively ferment succinate into propionate, thus potentially influencing intestinal health and overall metabolism [69,70]. Through these results supported by previous studies, it is estimated that yaks can adapt to different altitudes and temperatures by modifying these bacteria.

The gut microbiota plays a crucial role in various aspects, including digestion and nutrient absorption, immune regulation, metabolic control, defense against pathogens, impact on the nervous system, and hormone regulation [71]. Therefore, this study further investigates the metabolic functions of the gut in light of these considerations. In the bacterial KEGG functional prediction analysis, the relative abundance of the digestive system compromised the majority. Combined with COG function predictions, yaks living in Nyêmo county possess enhanced capabilities in inorganic ion transport, lipid transport, and metabolism, as well as RNA processing and modification. Enhanced inorganic ion transport capability may contribute to maintaining ion balance inside and outside cells, playing a crucial role in adapting to the unique climatic conditions and potential oxygen scarcity in high-altitude regions [72]. Lipids may serve as an efficient source of energy in high-altitude environments and the strengthened lipid transport functions provide additional energy reserves to adapt to cold climates [73]. The enhanced ability for RNA processing and modification may involve more flexible gene expression and adaptability, thus aiding yaks in more effectively coping with temperature fluctuations and other survival challenges in high-altitude environments [74].

In the fungal COG functional prediction analysis, yaks living in Damxung county had a higher relative abundance of lipid transport and metabolism than other groups, whereas yaks living in Linzhou county exhibited the highest relative abundance of secondary metabolite biosynthesis transport and catabolism as well as inorganic ion transport and metabolism. Furthermore, yaks residing in Nyêmo county displayed the highest relative abundance of translation ribosomal structure and biogenesis, cell cycle control, cell division, chromosome partitioning, replication, recombination and repair, nucleotide transport, and metabolism. The ability of cell cycle control and division is associated with variations in cellular processes adapting to different climatic conditions [75]. The transport and metabolism of nucleotides, which play a role in cellular biology and gene expression regulation, might have an impact on adapting to temperature and climate changes [76]. These changes indicate that the adaptability of yaks to different altitudes and temperatures has an impact on alterations in their metabolic functions.

## 5. Conclusions

Nexus to the above, this study illuminates the significant influence of altitude and temperature on the gut microbiota of Tibetan yaks. Through detailed sequencing analyses, it was found that different microbial communities are present in yaks from different altitudes with key differences in bacterial phylogenetic diversity and fungal richness. The findings highlight the presence of specific microbial genera and metabolic functions that are indicative of yaks’ ability to adjust to diverse climatic conditions. This highlights the importance of the gut microbiome in the physiological adaptation of yaks to high altitudes.

## Figures and Tables

**Table 1:** The sequence information of each sample.

Sample	Input	Filtered	Effectie (%)	AvgLen (bp)	GC (%)
DXS1	149055	142514	95.61	226.85	53.405
DXS2	141336	135362	95.77	226.4	53.23
DXS3	145569	139397	95.76	225.95	53.295
DXS4	145326	139083	95.7	227.35	53.285
DXS5	135439	129753	95.8	226.95	53.735
DXS6	140421	134845	96.03	226.3	53.725
LZS1	148314	142163	95.85	226.8	53.44
LZS2	135054	129488	95.88	226.4	52.755
LZS3	139428	133871	96.01	225.8	53.24
LZS4	144026	138466	96.14	227.4	53.18
LZS5	142422	136824	96.07	226.85	53.33
LZS6	136476	131350	96.24	226.3	53.655
NMS1	142805	136477	95.57	225.85	53.59
NMS2	141404	135608	95.9	227.45	53.67
NMS3	143945	137735	95.69	226.95	53.53
NMS4	136989	131526	96.01	226.3	52.86
NMS5	139503	133409	95.63	225.85	52.98
NMS6	138607	132501	95.59	227.4	53.275

## Data Availability

The raw data in this study were deposited in the NCBI database under accession number: PRJNA1097821.
